# Biomonitoring of Deoxynivalenol and Deoxynivalenol-3-glucoside in Human Volunteers: Renal Excretion Profiles

**DOI:** 10.3390/toxins11080466

**Published:** 2019-08-08

**Authors:** Marcel Mengelers, Marco Zeilmaker, Arnau Vidal, Marthe De Boevre, Sarah De Saeger, Rudolf Hoogenveen

**Affiliations:** 1 National Institute for Public Health and the Environment, Department of Food Safety, NL-3721 Bilthoven, The Netherlands; 2 Laboratory of Food Analysis, Department of Bioanalysis, Faculty of Pharmaceutical Sciences, Ghent University, B-9000 Ghent, Belgium; 3 National Institute of Public Health and the Environment, Department of Statistics, Informatics and Mathematical Modelling, NL-3721 Bilthoven, The Netherlands

**Keywords:** modified deoxynivalenol, DON, biomonitoring, renal excretion, kinetic modelling

## Abstract

Biomarkers for the determination of the dietary exposure to deoxynivalenol (DON) have been proposed in the past but so far no quantification of their use in humans has been carried out. Following a human intervention study with two mycotoxins, namely DON and deoxynivalenol-3-glucoside (DON3G), the renal excretion of these compounds, including their phase II metabolites, was analysed. The purpose was to develop biokinetic models that can be used to determine: (1) the preferred (set of) urinary biomarker(s), (2) the preferred urinary collection period, and (3) a method to estimate the dietary exposure to these mycotoxins. Twenty adult volunteers were restricted in consuming cereals and cereal-based foods for 4 days. At day 3, a single dose of 1 µg/kg body weight of DON or DON3G was orally administered to 16 volunteers; 4 volunteers served as control. All individual urine discharges were collected during 24 h after administration. The metabolism and renal excretion could be described by a biokinetic model using three physiological compartments (gastrointestinal tract, liver, and kidneys). Kinetic analysis revealed a complete recovery of the renal excretion of total DON (mainly DON and its glucuronides) within 24 h after administration of DON or DON3G. The so-called ‘reverse dosimetry’ factor was used to determine the preferred (set of) biomarker(s) and to estimate the dietary intake of the parent compounds in the future. The fact that DON3G was absorbed and mainly excreted as DON and its glucuronides confirms that DON3G (as well as other modified forms) should be taken into account in the exposure and risk assessment of this group of mycotoxins.

## 1. Introduction

Mycotoxins are substances produced by micro-fungi growing on plants and derived products during their production and storage. The production of mycotoxins depends on the plant species itself, the fungal species, temperature, and humidity. Deoxynivalenol (DON) is a foodborne mycotoxin that is primarily produced by *Fusarium* fungi. DON frequently occurs in grains and grain-based products [1]. DON-3-glucoside (DON3G) is a modified form of DON (also called masked DON) and is the main plant metabolite of DON [2,3]. Other forms of DON have been reported, like 3-acetyldeoxynivalenol (3-ADON) and 15-acetyldeoxynivalenol (15-ADON) [4].

DON is also known as vomitoxin and this trivial name is related to the fact that emesis is the most sensitive functional manifestation of acute toxicity in the pig, dog and cat after either oral or parenteral administration [4]. DON has been involved in a number of incidents of human intoxication in Asia and its acute emetic effects in humans are similar to those in animals. The most common effects of long-term dietary exposure of animals to DON are weight gain suppression and anorexia. In 2011, the Joint FAO/WHO Expert Committee on Food Additives (JECFA) has reported that the acetylated derivative 3-ADON was rapidly and extensively deacetylated to DON in animals [4]. In 2017, the European Food Safety Authority (EFSA) noted that 3-ADON and 15-ADON were largely deacetylated to DON prior to systemic distribution, such that they might induce the same acute and chronic effects as DON. EFSA also stated that DON3G could be cleaved to DON by bacteria in the gastrointestinal tract and distributed, metabolised and excreted similarly to DON [5].

EFSA has established a tolerable daily intake (TDI) of 1 µg/kg body weight (bw)/day for the sum of DON, DON3G, 3-ADON, and 15-ADON, a so-called group-TDI [5]. The estimated mean chronic dietary exposure in Europe was above the group-TDI in infants, toddlers and other children, and at high exposure also in adolescents and adults, indicating a potential health concern. The most important contributors to the chronic dietary exposure to DON, DON3G, 3-ADON, and 15-ADON are grain-based products, especially ‘bread and rolls’, ‘fine bakery wares’, and ‘pasta (raw)’. In order to assess acute human health risk, epidemiological data from ycotoxicosis were assessed, and a group acute reference dose (ARfD) of 8 µg/kg bw per eating occasion was calculated. Estimates of acute dietary exposures were below this dose and did not raise a health concern in humans [5].

A study in three European countries (Italy, Norway, and the United Kingdom) provided data on levels of total DON (free DON plus (unidentified) glucuronides) and de-epoxy-deoxynivalenol (DOM-1) in human urine samples from different population groups [6]. Morning urine samples were collected over two consecutive days from 635 volunteers and associated food consumption was recorded on the same days. On average, DON glucuronides accounted for 66–80% of the total DON in urine. The remaining portion was found as free DON. DOM-1 was detected in low concentrations in 12% of the Norwegian samples, in 1.5% of the Italian samples, and in none of the samples from the UK. It was concluded that de-epoxidation did not represent a main metabolic path in the excretion route of ingested DON. EFSA concluded that the exposure estimates, derived from the biomarker data from these three European countries, were of the same order of magnitude as the exposure estimates for the sum of DON, 3-ADON, 15-ADON, and DON3G derived from the occurrence data reported to EFSA and the dietary surveys from these countries [5].

So far, only one study has been reported where urine samples were obtained from one human volunteer following a diet, naturally contaminated with DON and some of its modified forms, over a period of four days [7]. These data suggest that approximately 70% of ingested DON is excreted via urine, of which about 80% was in conjugated forms, mainly as DON-15-glucuronide (DON-15-GlcA) that was about threefold more efficiently formed than DON-3-glucuronide (DON-3-GlcA). However, a difference between the renal clearance of DON and DON3G could not be made. Therefore, EFSA recommended well-designed quantitative studies on DON urinary excretion in different human sub-population groups to enable the use of DON biomarkers for human exposure assessments.

There is evidence that 3-ADON and 15-ADON are largely deacetylated prior to systemic distribution [4,5]. Whether DON3G is hydrolysed to DON by bacteria and/or enzymes, present in the human gut, prior to absorption and distribution of DON, and/or that part of DON3G is absorbed as glucoside remains unclear. Studies in pigs after oral administration of DON3G are also not univocal. In one study [8] DON3G was found in urine after oral administration but in another study DON3G could not be detected in systemic plasma nor in vena porta plasma after oral administration of DON3G [9].

Data on the fate of other investigated dietary glycosides like the flavonoid glycosides are also not conclusive regarding the form that is absorbed (aglycone or glycoside) in humans. However, a rapid and almost complete conversion of the flavonoid glucosides to glucuronides, which in turn are being distributed in circulating blood and excreted in urine, has been observed by many authors [10,11].

Therefore, this study was designed to unravel the renal excretion of DON and DON3G in humans after single-dose administration. A human intervention study was performed with 20 adult volunteers (average age 32 years, 55% women and 45% men) [12]. An oral single dose of DON or DON3G was administered at the level of the group-TDI, namely 1 µg/kg bw, to 16 volunteers while 4 subjects served as controls. Two days prior to and two days after single-dose administration, the volunteers followed a diet that did not contain grains and grain-based products. All individual urine discharges were collected during 24 h and samples were analysed by liquid chromatography coupled to tandem mass spectrometry (LC-MS/MS) for DON, DON3G, 3-ADON, 15-ADON, DOM-1, DON-3-GlcA, and DON-15-GlcA.

Subsequently, biokinetic models were developed to analyse the renal excretion of these compounds, including their phase II metabolites. These models were used to determine: (1) the preferred (set of) urinary biomarker(s), (2) the preferred urinary collection period, and (3) a method to estimate the dietary exposure to these mycotoxins by means of biomonitoring.

## 2. Results

### 2.1. General Results

The (raw) urinary data were obtained from a human intervention study published by Vidal et al. [12]. Urinary concentrations of DON and its conjugates DON-3-GlcA and DON-15-GlcA after single dose administration of DON were above the quantification limit for most volunteers until the end of the first experiment. Urinary concentrations of DON3G after single dose administration of DON3G were above the quantification limit for most volunteers during the first two or three collection intervals. Twelve hours after administration DON3G was not detectable in any of the volunteers. Urinary concentrations of DON and its conjugates DON-3-GlcA and DON-15-GlcA, after single-dose administration of DON3G, were above the quantification limit for most volunteers until the end of the second experiment [12].

The main metabolite observed in urine after administration of DON and DON3G was DON-15-GlcA. Moreover, in both experiments, the excreted amounts of DON-15-GlcA were always higher than for DON and DON-3-GlcA. The absorption, distribution, metabolism, and excretion (ADME) of DON and DON3G was a rapid process because after 24 h more than 99% of total DON excreted (sum of DON and its glucuronides), or total DON3G (sum of DON3G, DON and its glucuronides) was recovered. After DON3G administration, the parent compound appeared at its maximum amount in the first voided urine sample of all volunteers. Apparently, part of DON3G is rapidly absorbed and renally excreted.

After administration of DON, approximately 20–30% of the total DON recovered in urine during 24 h was identified as the parent compound. Neither DON3G nor DOM-1 was detected in urine. After administration of DON3G approximately 5–10% of total DON3G recovered in urine, was the parent compound and approximately 7% was DOM-1 [12]. Despite the fact that DON3G was only detected in urine during the first hours after administration of DON3G, the observation that (a small) part of the parent compound was absorbed and excreted unchanged made it necessary to develop other models describing the kinetics of DON3G than the one used for DON.

### 2.2. Deoxynivalenol (DON) Kinetics

#### 2.2.1. Model Selection

**Figure 1:**
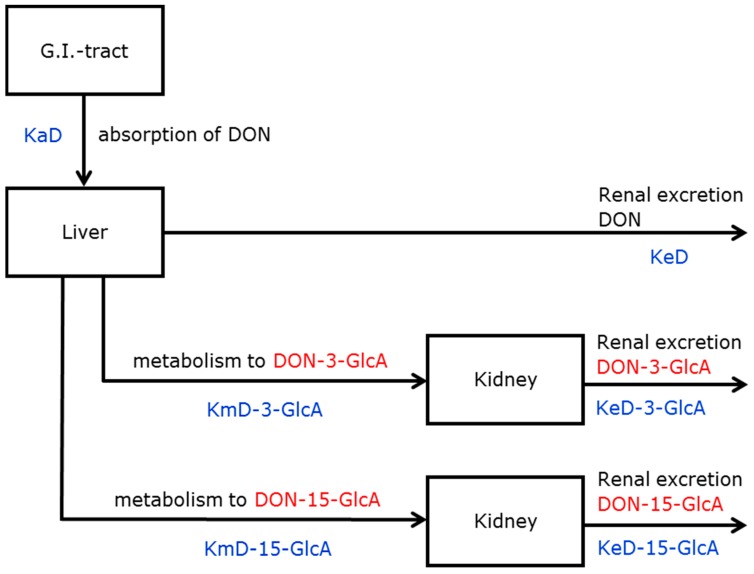
Model I: extended model for the metabolism and excretion of DON and two glucuronides. DON = deoxynivalenol, KaD = absorption rate constant of DON, KeD = excretion rate constant of DON, KmD-3-GlcA = metabolic rate constant of DON-3-GlcA, KeD-3-GlcA = excretion rate constant of DON-3-GlcA, KmD-15-GlcA = metabolic rate constant of DON-15-GlcA, KeD-15-GlcA = excretion rate constant of DON-15-GlcA.

After single-dose administration of DON, taking all dosed volunteers into account, it was possible to describe the renal excretion of DON, DON-3-GlcA, and DON-15-GlcA with a simple model (model A, Figure S1 in Supplementary Materials) as well as with an extended model (model I, Figure 1). However, using the simple model, some disadvantages were observed (data not shown): (i) negative values were obtained for some rate constants, (ii) confidence intervals of parameters were larger than for model I, (iii) correlation between parameters were larger than for model I, and (iv) the Akaike Information Criterion (AIC) was larger than for model I. Therefore, the extended model I was used to calculate the fraction absorbed (Fabs) and the kinetic rate constants.

#### 2.2.2. Parameter Estimation

Fabs and the rate constants in model I were modelled as fixed and random effects. The results of the parameter estimations modelled as fixed effects are given in Table 1. Random effect parametrisation had only an added value for Fabs and the absorption rate constant of DON (data not shown). The excretion rate constant of DON-15-GlcA had the highest coefficient of variation (CV), namely 20%. The CV of Fabs and the other rate constants varied from 10% to 13%.

**Figure 2:**
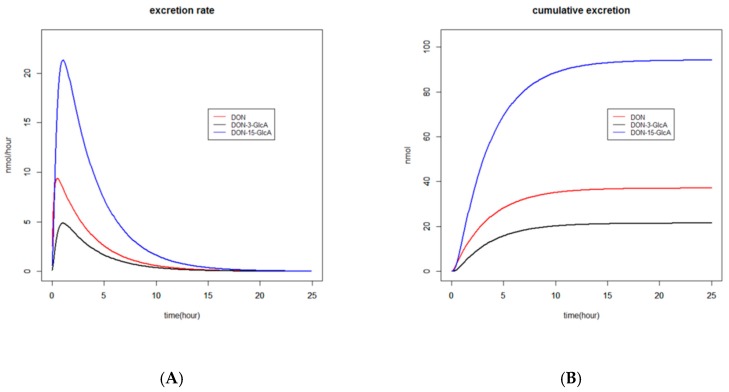
Time course of estimated renal excretion of DON, DON-3-GlcA, and DON-15-GlcA after single-dose administration of DON to 16 volunteers. Excretion rates (in nmol/hour) and cumulative amounts (in nmol) are shown in left (**A**) and right (**B**) plot, respectively.

The time to reach the maximum excreted amount (Tmax) of DON, DON-3-GlcA, and DON-15-GlcA in urine, based on the estimated parameters, varied between 0.5 and 1 h. This can also be observed in Figure 2A, where the estimated excretion rates of DON, DON-3-GlcA, and DON-15-GlcA versus time are given.

#### 2.2.3. Graphs

The time courses of the estimated renal excretion of DON, DON-3-GlcA, and DON-15-GlcA are presented in Figure 2 in two different manners. In Figure 2A, the excretion rates (in nmol/hour) are shown and Tmax for the various substances is visualised. In Figure 2B, the cumulative amounts (in nmol) are shown and the time to reach complete excretion is visualised. The ratio of DON-15-GlcA/DON and DON-15-GlcA/DON-3-GlcA after 24 h was respectively 2.6 and 4.5.

In Figure S4.1 in the Supplementary Materials, the actual cumulative amounts (in nmol) of DON-15-GlcA in all individuals after DON administration are shown.

#### 2.2.4. Recovery

When at the end of the experiment the total recovery was calculated based on the molar amounts recovered for DON, DON-3-GlcA, and DON-15-GlcA, a recovery of 64% ± 22% was obtained. Among the volunteers the percentage absorbed varied from 30% to 98%. The discrepancy between estimated Fabs (0.56) and the measured total recovery can be explained by the difference in fitting a more complex compartmental model to all data and calculating a recovery based on intake and the cumulative excretion data only.

### 2.3. DON-3-glucoside (DON3G) Kinetics

#### 2.3.1. Model Selection

**Figure 3:**
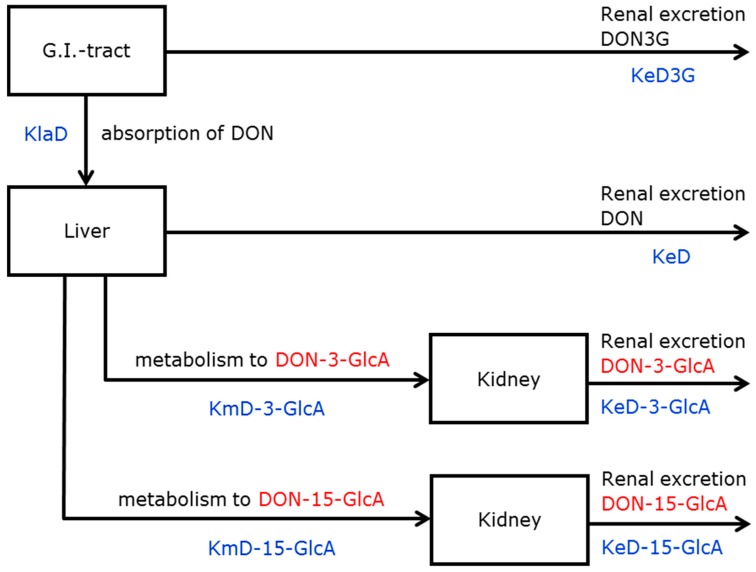
Model II: absorption and excretion of DON3G combined with hydrolysis of DON3G in the G.I.-tract and subsequent metabolism and excretion of DON. DON = deoxynivalenol, KlaD = lumped absorption rate constant of DON (including hydrolysis of DON3G to DON), KeD3G = excretion rate constant of DON3G, KeD = excretion rate constant of DON, KmD-3-GlcA = metabolic rate constant of DON-3-GlcA, KeD-3-GlcA = excretion rate constant of DON-3-GlcA, KmD-15-GlcA = metabolic rate constant of DON-15-GlcA, KeD-15-GlcA = excretion rate constant of DON-15-GlcA.

After administration of DON3G, the renal excretion of the parent compound (a glycoside) was faster than the aglycone (DON) and its glucuronides. This observation had two consequences for the model applied for DON3G: (1) the rapid renal excretion of DON3G did not allow including an absorption rate constant for DON3G because absorption and distribution of DON3G appeared to be instantaneous and (2) the absorption of DON was a lumped process (absorption of DON including the hydrolysis of DON3G to DON). Consequently, models comprising the physiological passage of DON3G through the gut wall and liver could not accurately describe the renal excretion of DON3G. These findings rejected models B and C shown in the Supplementary Materials (Figures S2 and S3). Therefore, the renal excretion of DON3G was modelled in such a way that gut wall and liver passage was circumvented. This resulted in the selection of model II, as depicted in Figure 3.

#### 2.3.2. Parameter Estimation

Similar to model I, Fabs and the rate constants were modelled as fixed and random effects. The results of the parameter estimations modelled as fixed effects are given in Table 2. Again, random effect parametrisation had only an added value for Fabs and the lumped absorption rate constant of DON (including hydrolysis of DON3G to DON) (data not shown). Fabs had the smallest CV (8%), followed by the excretion rate constants of DON-3-GlcA and DON-15-GlcA (both 14%). The other rate constants had much higher CV due to the fact that they had large confidence intervals and/or were highly correlated (data not shown). 

**Figure 4:**
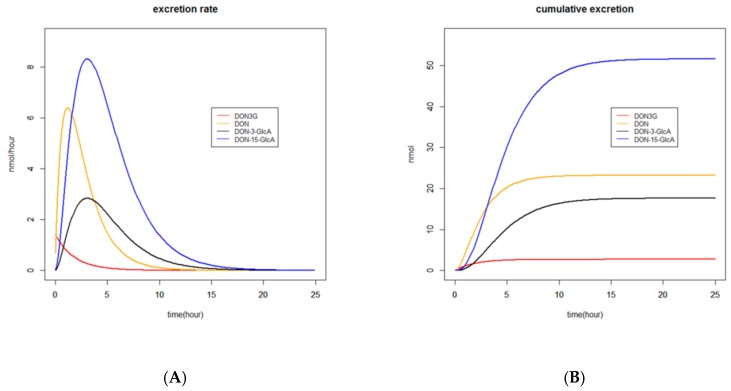
Time course of estimated renal excretion of DON3G, DON, DON-3-GlcA, and DON-15-GlcA after single-dose administration of DON3G to 16 volunteers. Excretion rates (in nmol/hour) and cumulative amounts (in nmol) are shown in left (**A**) and right (**B**) plot, respectively.

The renal excretion of DON3G was too fast to be able to determine the time to reach the maximum excreted amount (Tmax). Tmax of DON, DON-3-GlcA, and DON-15-GlcA, based on the estimated parameters, varied between 1 and 3 h. This can also be observed in Figure 4A where estimated excretion rates of DON3G, DON, DON-3-GlcA, and DON-15-GlcA versus time are given.

#### 2.3.3. Graphs

The time courses of the estimated renal excretion of DON3G, DON, DON-3-GlcA, and DON-15-GlcA are presented in Figure 4 in two different manners. In Figure 4A, the excretion rates (in nmol/hour) are shown and Tmax for the various substances is visualised. In Figure 4B the cumulative amounts (in nmol) are shown and the time to reach complete excretion is visualised. The ratio of DON-15-GlcA/DON and DON-15-GlcA/DON-3-GlcA after 24 h was respectively 2.2 and 3.0.

In Figure S4.2 in the Supplementary Materials, the actual cumulative amounts (in nmol) of DON-15-GlcA in all individuals after DON3G administration are shown.

#### 2.3.4. Recovery

When at the end of the experiment the total recovery was calculated based on the molar amounts recovered for DON3G, DON, DON-3-GlcA, and DON-15-GlcA, a recovery of 58% ± 16% was obtained. Among the volunteers, the percentage absorbed varied from 29% to 84%. The discrepancy between estimated Fabs (0.53) and the measured total recovery is smaller than for DON.

### 2.4. Biomonitoring

#### 2.4.1. Preferred Biomarker and Reversed Dosimetry Factor

To calculate the intake of the parent substance the Reversed Dosimetry Factors (RDFs, see Materials and Methods section) of DON, DON-3-GlcA, DON-15-GlcA, and total DON (sum of DON and its glucuronides) were used. These RDFs and their confidence intervals are given in Table 3. It can be concluded that after single-dose administration of DON, the confidence intervals of DON-15-GlcA and total DON are comparable (respectively 6.6 and 6.1) and both are lower than for DON or DON-3-GlcA. The reversed dosimetry factor for DON-15-GlcA will apply to smaller urinary amounts than for total DON and using the RDF for DON-15-GlcA will lead to a smaller confidence interval for the estimated exposure to DON. Therefore, the preferred biomarker for the estimation of DON exposure is DON-15-GlcA and the RDF to be used is 2.7.

After single-dose administration of DON3G, the confidence intervals of DON-15-GlcA and total DON are 5.4 and 2.8, respectively. Again both confidence intervals are smaller than for DON or DON-3-GlcA. In this case it is difficult to have a distinct preference for DON-15-GlcA or total DON for the estimation of DON3G exposure. The RDF for DON-15-GlcA is 3.4 and for total DON 1.7.

#### 2.4.2. Collection Period

The so-called delta method (see Materials and Methods section) was used to calculate how long it takes to reach 95% of the cumulative amount excreted of each substance and total DON at the end of each experiment. Regardless of the substance these periods (± standard error of mean) were respectively 12.0 ± 0.5 and 11.7 ± 1.0 h for model I and II, respectively (Figure 2 and Figure 4).

## 3. Discussion

### 3.1. Absorption, Distribution, Metabolism and Excretion (ADME) of DON and DON3G

#### 3.1.1. Absorption and Distribution

Based on the renal excretion profiles, we conclude that DON as well as DON3G are rapidly absorbed and distributed in humans. Moreover, based on the ratio of the total amount excreted after 24 h and the dose, the percentage of DON and DON3G absorbed is comparable: respectively 64% ± 22% and 58% ± 16%. After oral administration of DON or DON3G, the maximum percentage absorbed in the volunteers was 98% for DON and 84% for DON3G. This indicates that in some volunteers recovery of total DON was almost complete.

Previously to this study, there were no quantitative data on the absorption and bioavailability of DON and DON3G in humans [13]. Therefore, a comparison is made with the absorption and bioavailability of other dietary glycosides, namely the flavonoid glycosides. Compared to DON3G, some of these flavonoid glycosides possess similar molar masses and O-glycosidic bonds. It has been shown that the type and position of the attached sugar make a critical difference to both the site and extent of absorption [14,15,16,17,18]. In general, studies in humans show that absorption, hydrolysis of the mono O-glucoside, subsequent metabolism to various glucuronides and excretion is relatively fast because maximum concentrations in plasma (and urine) are observed within a few hours [10,11,14]. However, it is not always clear whether the glucoside itself and/or the glucuronides are present at Tmax because plasma and urine samples were deconjugated with different enzymes before analysis and no distinction could be made between the glycosides and phase II metabolites like glucuronides and/or sulphates. Nowadays, LC/MS-MS methods enable the analysis of aglycone, glycoside(s), and phase II metabolite(s) without deconjugation. These new methods have been applied in studies with mono O-glucosides of flavonoids, like malvidin and hesperetin [14,15]. It was shown that these glucosides are rapidly and efficiently absorbed in the small intestine. Although hydrolysis of DON3G in the liver (after gut wall passage) cannot be ruled out, Berthiller et al. pinpointed that in vitro incubation with human cytosolic beta-glucosidases did not hydrolyse DON3G [19]. Based on short Tmax values observed in plasma and/or urine, our study, as well as the studies on the flavonoid glucosides, show that the absorption of these glucosides (predominantly after hydrolysis) more likely occurs in the upper part of the intestines (duodenum and proximal jejunum) and not in the colon. Therefore, enzymatic hydrolysis of DON3G in enterocytes by glucosidases and/or lactases is more likely to occur in humans than hydrolysis of glucosides by bacteria in the colon.

#### 3.1.2. Metabolism

Regarding the metabolism of DON and DON3G, our study confirmed earlier observations in humans that the main metabolites are the glucuronides DON-3-GlcA and DON-15-GlcA. A study in three European countries provided data on levels of total DON (free DON plus (unidentified) glucuronides) and DOM-1 in human urine samples from different population groups [6]. Morning urine samples were collected over two consecutive days from 635 volunteers and, on average, DON glucuronides accounted for 66% to 80% of the total DON in urine. The remaining portion was found as free DON. In a Belgian biomonitoring study, multiple mycotoxins were analysed in morning urine samples of 155 children and 293 adults [20]. DON-15-GlcA was the main urinary DON biomarker and was found in all urine samples in the ng/mL range. Somewhat lower levels of DON-3-GlcA were quantified in 91% of the urine samples collected from children and in 77% of the samples collected from adults. In turn, DON levels were lower than the levels of each of the DON glucuronides. Furthermore, Turner et al. have shown in several studies that the main metabolites of DON in humans are the glucuronides [21,22,23]. In a metabolic study conducted in one human volunteer it was shown that DON-glucuronides constituted 76% (range 72–80%) of the total DON consumed [7]. DON-15-GlcA was the main conjugation product, constituting 73% of the total DON-glucuronides while DON-3-GlcA constituted only 27%.

#### 3.1.3. Excretion

In our study, it was shown that DON as well as DON3G were rapidly and efficiently excreted renally. After oral administration of DON, the Tmax values observed in urine for DON, DON-3-GlcA, and DON-15-GlcA vary from 0.5 to 1 h. After oral administration of DON3G, the Tmax values observed in urine for DON, DON-3-GlcA, and DON-15-GlcA vary from 1 to 3 h. The fact that DON3G was partly present as a parent compound in urine of all volunteers during the first hours after administration shows that the glucoside was absorbed, distributed, and excreted very efficiently. In all volunteers, the maximum amount of DON3G was excreted in the first voided urine sample and consequently Tmax of DON3G could not be determined. Although the total amount excreted was only 1–5% of the administered dose, this observation could not be neglected, and it dictated the type of model that was selected. In the intervention study published by Vidal et al., it was shown for the first time that humans absorb and excrete DON3G unmetabolised [12]. Renal clearance of DON3G has also been observed after oral administration of a high, single dose of DON3G (116 µg/kg bw) to pigs [8]. In this study, approximately 2.5% of the dose was excreted during the first 8 h and another 1% of the dose during the rest of the 24-hour urine collection period. However, in a recent study in pigs, DON3G could not be detected in systemic plasma nor in vena porta plasma after oral administration of a single dose of DON3G (56 µg/kg bw) [9]. This result suggests a complete presystemic hydrolysis of DON3G in pigs.

To summarise, it was concluded that a minor part of DON3G (< 5% of the oral dose of DON3G) is absorbed as glucoside, and the major part (approximately 50% of the oral dose of DON3G) is absorbed as DON (after presystemic hydrolysis of DON3G), which in turn is renally excreted and metabolized to DON-3-GlcA and predominantly to DON-15-GlcA before excretion.

### 3.2. Modelling

#### 3.2.1. Model Selection

In the case of DON, a one compartment model, with intake being the inflow of DON and excretions being the outflows of DON, DON-3-GlcA, and DON-15-GlcA, was not able to describe the data accurately. Therefore, this model was rejected and a more complex model that included the main (lumped) physiological processes, i.e., absorption, distribution, metabolism, and excretion was investigated. This model contained three physiological compartments, i.e., gastrointestinal (G.I). tract, liver and kidneys, with related model variables being the amounts of DON in the G.I. tract and the liver, and the amounts of DON-3-GlcA and DON-15-GlcA in the kidneys, respectively. Distinguished transitions were between G.I. tract and liver (to be interpreted as absorption and transport of DON) and between liver and kidney (metabolism to DON-3-GlcA and to DON-15-GlcA and transport). Finally, outflow occurred from kidney (urinary excretion of DON, DON-3-GlcA, and DON-15-GlcA). Broekaert et al. could also not describe the toxicokinetics of DON after oral administration to pigs with a one compartment model and used a two compartment model instead (applying a central and a peripheral compartment) [9].

In the case of DON3G, at first, the compartmental model of DON was used for the absorption of DON3G (without prior hydrolysis) assuming hydrolysis of DON3G to DON to occur in the liver. Secondly, a model was tested in which three processes occur in the G.I. tract: absorption of DON3G, hydrolysis of DON3G to DON, and (subsequent) absorption of DON. However, both models were not able to describe the high initial excretion of DON3G. Therefore, the DON-model was extended with an extra transition, i.e., outflow of DON3G from the G.I. tract compartment. Note that the transition from the G.I. tract to the liver compartment should be interpreted differently in the DON3G-model: not only absorption and transport, but also presystemic hydrolysis. This presystemic hydrolysis was also observed by Broekaert et al. after oral administration of DON3G to pigs [9]. In contrast, in pigs this presystemic hydrolysis of the absorbed fraction was complete because no DON3G was detected in portal plasma.

#### 3.2.2. Parametrisation

After selecting the model structure, its parametrisation was performed by statistically defining the model parameters to be distinguished. As the cumulative excretion amounts differed largely between individuals, it was investigated to what extent this heterogeneity could be incorporated in the model by assuming its parameters to differ between individuals. Or, in other words, a selection was made which parameters could be assumed to be different across individuals (random parameters) and which to be equal (fixed parameters). Clearly, assuming the fraction of absorbed DON and DON3G to be random (while assuming all other parameters fixed) sufficed to cover the mentioned interindividual heterogeneity. This heterogeneity in combination with the (relatively) small number of volunteers, did not allow differentiating men and women.

### 3.3. Biomonitoring

#### 3.3.1. Preferred Biomarker and Exposure Calculation

Based on the RDFs of DON, DON-3-GlcA, DON-15-GlcA, total DON, and a desired small confidence interval for the estimated exposure to DON and DON3G, the preferred biomarker for the estimation of DON and DON3G exposure is DON-15-GlcA. If, for analytical reasons (see below), DON-15-GlcA in urine cannot be measured, then total DON (sum of urinary DON and glucuronides) is preferred as second best by using enzymatic hydrolysis (deglucuronidation) of urine samples.

In practice, simultaneous exposure to DON and DON3G will occur in varying degrees, depending on the type of food product. For example, Janssen et al. have estimated the DON3G/DON ratio for several food product categories based on literature data on co-occurring levels of DON and DON3G [24]. This resulted in a DON3G/DON ratio of 0.2 in grains and grain-milling products, 0.3 in grain-based products, and 0.8 in beer. It should also be noted that our calculations are based on single-dose administration of DON and DON3G on an empty stomach and an effect of the food matrix on the bioavailability cannot be ruled out. Another practical point is that not all analytical laboratories have the facilities to determine individual glucuronides because commercial sources for the standards of DON-glucuronides are non-existent and no (certified) reference materials are available for urinary DON-glucuronides. Therefore, in case of an exposure of a mixture of DON and DON3G (ratio unknown), we propose to use a RDF of 3 (range 2.7–3.4) based on the cumulated excretion of DON-15-GlcA and a RDF of 1.6 (range 1.5–1.7) based on cumulated excretion of total DON.

A metabolic study was conducted in one human volunteer who consumed a diet naturally contaminated with 138 µg DON, 20 µg 3-Ac-DON, and 7 µg DON3G for 4 days [7]. Urinary DON and conjugated DON were measured in urine samples collected over a period of 8 days including 2 days before and 2 days after the 4 exposure days. The daily excretion of total DON was on average 68% (range 60–73%) during the 4 exposure days and DON-glucuronides constituted 76% (range 72–80%) of the total DON recovered. The ratios of DON-15-GlcA, DON-3-GlcA, and DON-glucuronides to DON were fairly stable over the 8 days. In addition, DON-15-GlcA was the main conjugation product, constituting 73% of total DON-glucuronides (range 69–76%), while DON-3-GlcA constituted only 27% (range 24–31%). This means, roughly, that the RDF for DON-15-GlcA and DON-3-GlcA are respectively 2.7 and 7.2. Both RDFs are within the CI that we have calculated for the RDFs of DON-15-GlcA and DON-3-GLcA but please note that our CI for the RDF of DON-3-GlcA is (too) large. Nevertheless, the RDF of DON-15-GlcA calculated for this experiment corresponds very well with the RDF extrapolated from Warth et al. [7].

#### 3.3.2. Preferred Collection Period

On average, and regardless of the substance, it takes approximately 12 h to reach 95% of the cumulative amount excreted. For practical reasons, the preferred urinary collection period for the exposure to DON and/or DON3G would be 24 h. In case consumers are exposed to DON and/or DON3G during the previous day, including the evening meal, complete recovery would be achieved whenever the early-morning urine of the next day is included. Due to the rapid renal excretion of DON and DON3G, it is not recommended to use spot urine or early-morning urine samples to calculate the dietary exposure of DON and DON3G.

## 4. Conclusions

This is the first time that biokinetic models have been developed to describe the renal excretion of DON and DON3G in humans. Although a kinetic analysis based on (only) urine data has its limitations, these models enable us to determine the preferred (set of) urinary biomarker(s) (namely DON-15-glucuronide or total DON), the preferred urinary collection period (24 h), and to estimate the dietary exposure to these mycotoxins (by means of a reversed dosimetry factor). In order to evaluate these biokinetic models, it is proposed to conduct future research in cohorts, with a sufficient number of volunteers, in which DON exposure and renal excretion should be quantified.

## 5. Materials and Methods

### 5.1. Preparation of the DON/DON3G Dose

Anticipating a rapid renal excretion, the dose had to be sufficiently high to be able to determine urinary concentrations of DON, DON3G, and its metabolites quantitatively in urine during 24 h after dosing. The Ethical Committee of the Ghent University Hospital (B670201630414) approved a single-dose administration of DON as well as DON3G at the group-TDI level of 1 µg/kg bw [12]. The dose was given as an aqueous solution on an empty stomach.

### 5.2. Study Design

The human intervention study was conducted according to the guidelines laid down in the declaration of Helsinki, and was approved by the Ethical Committee of the Ghent University Hospital (B670201630414). The approval date was 8 December 2017. Informed consent was obtained from all individual participants included in the study. The details of the design of the human intervention study have been published by Vidal et al. [12].

In short, the study was performed with 20 healthy adults, including 11 women (55%) and 9 men (45%). Their mean age was 32 years, range 18–61 years. Two days prior to and two days after single-dose administration, the volunteers followed a strict diet that did not contain grains and grain-based products. On the third day in the morning, 16 subjects received an oral dose of DON or DON3G based on the TDI and their body weight. Four volunteers did not receive a bolus of DON or DON3G and served as controls. All individual urinary discharges were collected during 24 h, and stored at −20 °C until analysis. Samples were analysed by LC-MS/MS for DON, DON3G, 3-ADON, 15-ADON, DOM-1, DON-3-GlcA, and DON-15-GlcA.

### 5.3. Sample Preparation and Liquid Chromatography coupled to Tandem Mass Spectrometry LC-MS/MS Analysis

The details of the preparation of the urine samples and the targeted LC-MS/MS analysis have been published by Vidal et al. [12]. In short, the thawed urine sample was diluted with an acidic acetonitrile/water solution and centrifuged. An aliquot of the non-polar fraction was evaporated to dryness and redissolved in 250 µl injection solvent. These extracts were injected into an HPLC-MS/MS. The applied LC-MS/MS method was successfully validated based on the European Commission Decision 2002/657/EC laying down the rules for analytical methods to be used in the testing of official samples. DON, DON3G, 3-ADON, 15-ADON, DOM-1, and isotope-labelled (13C15) DON, used as internal standard, were obtained from a commercial source (Sigma Aldrich, Bornem, Belgium). DON-3-GlcA was supplied by Dr. Huybrechts (CODA-CERVA, Tervuren, Belgium).

### 5.4. Modelling

The first intervention study was carried out with a single dose of DON, while the second study was performed two months later with a bolus of DON3G and the kinetic models were developed in that order. All models were written in the programming language R, the source code is available upon request.

The data were analysed by fitting a compartmental model to the data using a standard non-linear fitting procedure. Because only data on the intake and excretion were available, the compartmental model has to be sufficiently complex to describe the physiological processes and simple enough to be statistically identifiable.

#### 5.4.1. Models for DON

The suitability of two models was tested for the description of the collected urinary data after administration of DON: a simple model and an extended model. The simple model is a 1-compartment model that comprises the absorption of DON and the elimination of DON, DON-3-GlcA, and DON-15-GlcA (shown as model A in Figure S1 in Supplementary Materials). It is a classical (toxico-) kinetic model in which the (one and only) compartment is not a physiologically based compartment but a mathematical presentation of the human body. This model includes 4 rate constants: one for the absorption of DON, one for the renal excretion of DON, and two lumped parameters for the elimination (metabolism and renal excretion) of DON-3-GlcA and DON-15-GlcA. The extended model (I) contains three physiological compartments (gastrointestinal tract, liver, and kidney) (Figure 1). This model includes the absorption and renal excretion of DON and two parallel processes that occur in the liver: the metabolism to and excretion of the two glucuronides. It includes 6 rate constants: one for the absorption of DON (KaD), two for the metabolism to DON-3-GlcA (KmD-3-GlcA) and DON-15-GlcA (KmD-15-GlcA), and three for the renal excretion of DON (KeD), DON-3-GlcA (KeD-3-GlcA), and DON-15-GlcA (KeD-15-GlcA). In addition, the fraction absorbed (Fabs) is also a parameter estimated in the model calculations.

#### 5.4.2. Models for DON3G

Based on the outcome of the first intervention study, several models were tested for the description of the collected urinary data after administration of DON3G. Eventually, model II was used which is based on the fact that the greater part of DON3G is hydrolysed to DON in the G.I. tract and subsequently, DON is absorbed, see Figure 3. The absorption, distribution, metabolism, and excretion (ADME) of DON in model II resembles model I and consequently, most rate constants have already been identified in the first intervention study, namely KeD, KmD-3-GlcA, KeD-3-GlcA, KmD-15-GlcA, and KeD-15-GlcA. In model II, a lumped absorption rate constant of DON (KlaD) that comprises the absorption of DON preceded by the presystemic hydrolysis of DON3G to DON was used. Compared to model I, one new excretion rate constant was added: the rate constant of the renal excretion of DON3G (KeD3G). Again, the fraction absorbed (Fabs) is also an estimated model parameter.

### 5.5. Calculations and Statistical Evaluations

All calculations were interval calculations, i.e., molar amounts of the investigated substances voided at different time intervals were used. Time courses of the absolute amounts excreted were used for the calculation of Fabs, the rate constants, and the time to reach the maximum excreted amount (Tmax) of the several compounds. Cumulative amounts of the excreted substances were not used for modelling but cumulative excretion plots are shown for presentation purposes. Cumulative amounts were used for the calculation of the preferred (set of) urinary biomarker(s) and the preferred urinary collection period.

The relative cumulative total excretion amounts differed largely between individuals and we modelled this heterogeneity by assuming the Fabs-parameter to be a random effect parameter. This means that this parameter is not fixed but differs between individuals according to a distribution to be estimated. The other model parameters are the transition rates between the compartments. The selections to be made are: which parameters are assumed to be different and which to be equal, and are these parameters fixed or random (in case the heterogeneity is insufficiently covered by the random Fabs-parameter).

Since the model is non-linear (the calculated amounts over time depend non-linearly on the transition rates) and parameters are random or fixed, we used the R-routine nlme (non-linear mixed effects) to fit the model. We assessed the best model parameterisation by using the Akaike Information Criterion (AIC) function that combines the model fit with the model complexity. The data values used were the time-interval excretion amounts, not the cumulative amounts. The reasons are twofold: (1) the cumulative amounts become unknown once a time-interval excretion amount is missing, and (2) successive cumulative amounts are correlated, and thus violate the model assumption of uncorrelated data values.

After assessing the best model fit in this way, we used the model results to calculate the time until a fixed proportion (95%) of the maximum amount was excreted. The calculation procedure applied was based on the so-called delta method [25]. Given the optimal parameter values and assuming the Fabs-parameter to be 1 (without any restriction), we calculated the time point when 95% of the intake was excreted. Next, we calculated the time points for one-at-a-time different parameter values. Finally, we approximated the variance of the time point by multiplying the co-variance matrix of the parameter estimations (output from the model fitting procedure) with the changes of the time points calculated above.

In order to determine the preferred (set of) biomarker(s) and the dietary exposure, the so-called ‘reverse dosimetry’ factor (RDF) was used. The reverse dosimetry factor is defined as the factor with which the cumulative excreted amount has to be multiplied to calculate the intake of the parent substance. Because the follow-up time was complete, meaning that no more DON or metabolites were expected to be excreted, we calculated the RDF as the ratio of the intake of DON or DON3G to the cumulative excreted amount. Therefore, we did not have to use the compartmental model to calculate the RDFs, but instead could use the data values on intake and cumulative excreted amounts of DON, DON-3-GlcA, DON-15-GlcA, and total DON (sum of DON and its glucuronides). Because the ratios are skewed distributed, we applied a logistic transformation to calculate the confidence bounds.

## Figures and Tables

**Table 1:** Estimations of the parameters (Fabs and rate constants) modelled as fixed effects belonging to model I (Figure 1) applied for the single dose administration of DON.

	Value	Standard Error
Fabs	0.56	0.06
KaD	0.31	0.04
KmD-3-GlcA	0.73	0.10
KmD-15-GlcA	3.29	0.39
KeD	1.28	0.14
KeD-3-GlcA	1.28	0.14
KeD-15-GlcA	2.31	0.47

Fabs = fraction absorbed, KaD = absorption rate constant of DON, KmD-3-GlcA = metabolic rate constant of DON-3-GlcA, KmD-15-GlcA = metabolic rate constant of DON-15-GlcA, KeD = excretion rate constant of DON, KeD-3-GlcA = excretion rate constant of DON-3-GlcA and KeD-15-GlcA = excretion rate constant of DON-15-GlcA.

**Table 2:** Estimations of the parameters (Fabs and rate constants) modelled as fixed effects belonging to model II (Figure 3) applied for the single dose administration of DON3G.

	Value	Standard Error
Fabs	0.53	0.04
KlaD	0.67	0.79
KmD-3-GlcA	0.13	0.15
KmD-15-GlcA	0.38	0.45
KeD3G	0.018	0.017
KeD	0.17	0.20
KeD-3-GlcA	0.49	0.07
KeD-15-GlcA	0.49	0.07

KlaD = lumped absorption rate constant of DON (including hydrolysis of DON3G to DON), KeD3G = excretion rate constant of DON3G

**Table 3:** Reversed dosimetry factors (RDFs), including confidence intervals (CIs), of separate substances and total DON after single dose administration of DON and DON3G.

Single Dose	Substance	RDF	CI	CI-lower Limit	CI-upper Limit
DON	DON	7.20	26.7	2.37	29.1
	DON-3-GlcA	12.6	40.2	4.12	44.34
	DON-15-GlcA	2.67	6.56	1.40	7.96
	total DON	1.45	6.07	1.03	7.10
DON3G	DON3G	-	-	-	-
	DON	6.95	11.47	3.53	15.00
	DON-3-GlcA	11.21	26.5	4.47	31.07
	DON-15-GLcA	3.37	5.35	1.90	7.25
	total DON	1.73	2.81	1.18	3.99

## Supplementary Materials

The following are available online at https://www.mdpi.com/2072-6651/11/8/466/s1, Figure S1: Model A: one-compartment model for the excretion of DON and two glucuronides, Figure S2: Model B: absorption of DON3G in the G.I.-tract and subsequent metabolism and excretion. Figure S3: Model C: absorption of a mixture of DON3G and DON in the G.I.-tract and subsequent metabolism and excretion, Figure S4: The actual cumulative amounts (in nmol) of DON-15-GlcA in all individuals and the best model fit after single, oral administration of DON (Figure S4.1) or DON3G (Figure S4.2).
