# Supplementary material for: Biomonitoring of Deoxynivalenol and Deoxynivalenol-3-glucoside in Human Volunteers: Renal Excretion Profiles
Source: Toxins (Basel). 2019 Aug 8;11(8):466. doi: 10.3390/toxins11080466 (PMC6723587; doi:10.3390/toxins11080466)
Supplement: Supplementary file 1 [file toxins-11-00466-s001.pdf]

Supplementary Materials: Biomonitoring of Deoxynivalenol and Deoxynivalenol-3-glucoside in Human Volunteers: Renal Excretion Profiles

Marcel Mengelers, Marco Zeilmaker, Arnau Vidal, Marthe De Boevre, Sarah De Saeger and Rudolf Hoogenveen

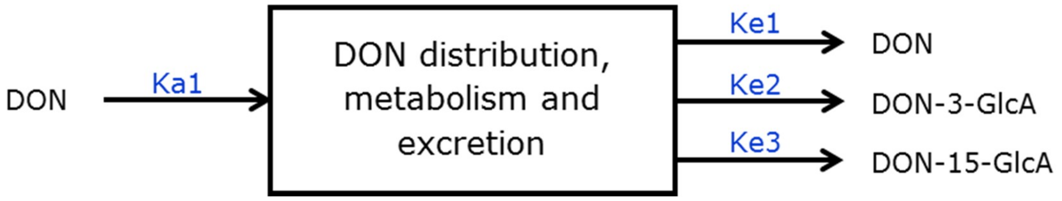

Figure S1. Model A: one-compartment model for the excretion of DON and two glucuronides. DON = deoxynivalenol, DON-GlcA= DON-Glucuronide, Ka1 = absorption rate constant of DON, Ke1 = elimination rate constant of DON, Ke2 = elimination rate constant of DON-3-GlcA, Ke3 = elimination rate constant of DON-15-GlcA. The elimination rate constants of the glucuronides are lumped parameters for the processes of metabolism and excretion.

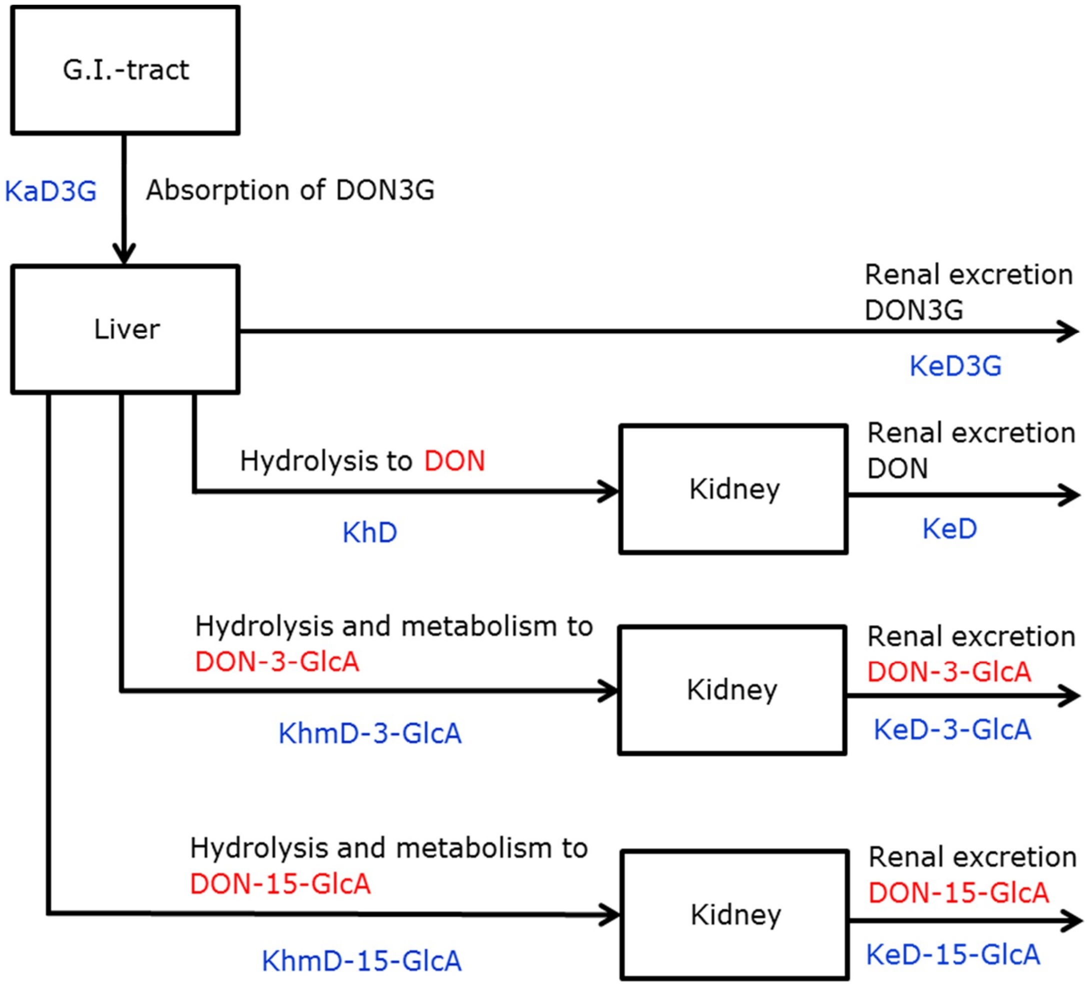

Figure S2. Model B: absorption of DON3G in the G.I.-tract and subsequent metabolism and excretion. DON3G = deoxynivalenol-3-glucoside, DON = deoxynivalenol, DON-GlcA= DON-Glucuronide, KaD3G = absorption rate constant of DON3G, KeD3G = excretion rate constant

of DON3G, KhD = hydrolysis rate constant of DON, KeD = excretion rate constant of DON, KhmD-3-GlcA = lumped (hydrolysis & metabolism) rate constant of DON-3-GlcA, KeD-3-GlcA = excretion rate constant of DON-3-GlcA, KhmD-15-GlcA = lumped (hydrolysis and metabolism) rate constant of DON-15-GlcA, KeD-15-GlcA = excretion rate constant of DON-15-GlcA.

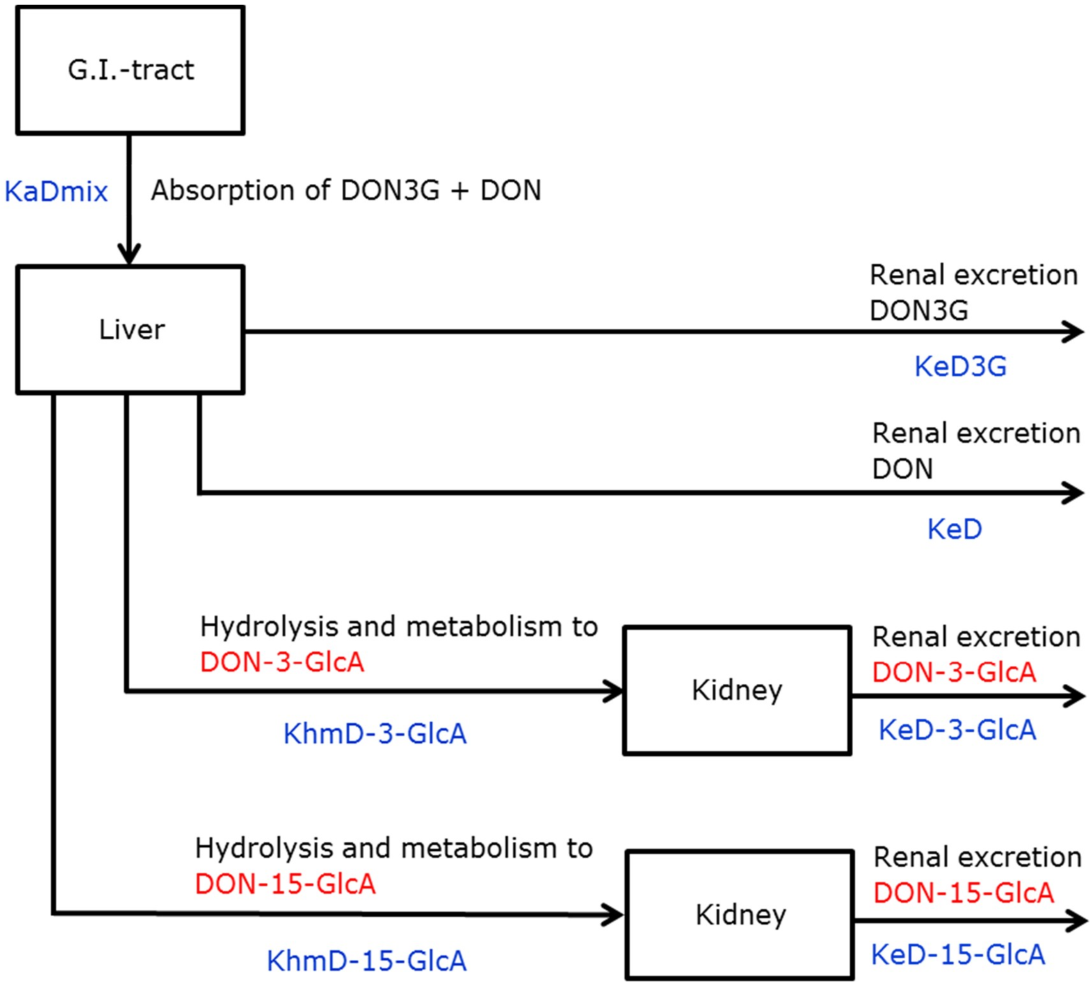

Figure S3. Model C: absorption of a mixture of DON3G and DON in the G.I.-tract and subsequent metabolism and excretion. DON3G = deoxynivalenol-3-glucoside, DON = deoxynivalenol, DON-GlcA= DON-Glucuronide, KaDmix = absorption rate constant of a mixture of DON3G and DON, KeD3G = excretion rate constant of DON3G, KeD = excretion rate constant of DON, KhmD-3-GlcA = lumped (hydrolysis & metabolism) rate constant of DON-3-GlcA, KeD-3-GlcA = excretion rate constant of DON-3-GlcA, KhmD-15-GlcA = lumped (hydrolysis and metabolism) rate constant of DON-15-GlcA, KeD-15-GlcA = excretion rate constant of DON-15-GlcA.

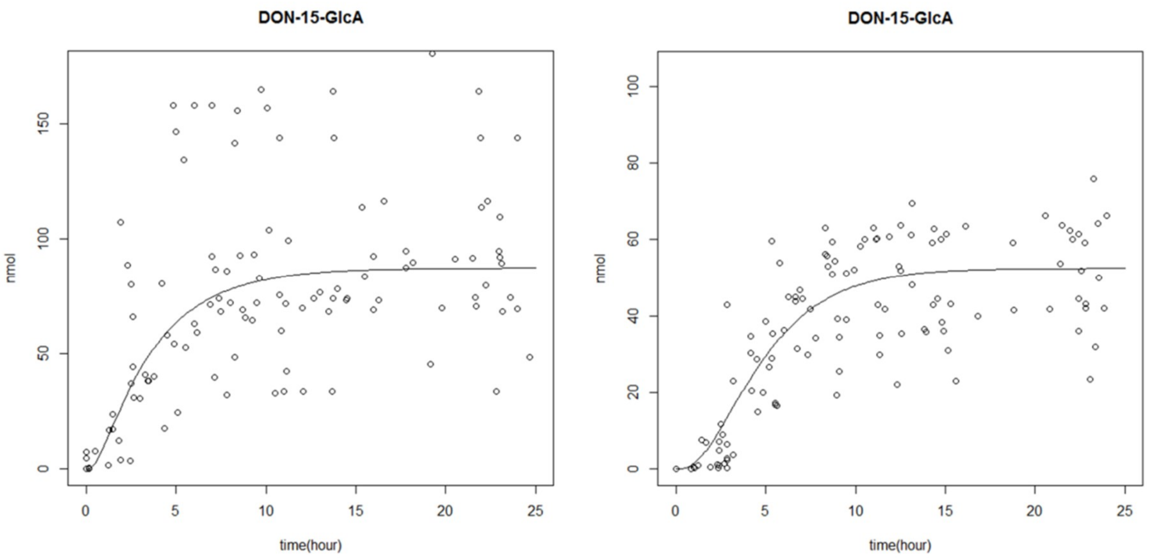

Figure S4. The actual cumulative amounts (in nmol) of DON-15-GlcA in all individuals and the best model fit after single, oral administration of DON (Figure **S4.1**, left plot) or DON3G (Figure **S4.2**, right plot).
